# Boron Deficiency in Trifoliate Orange Induces Changes in Pectin Composition and Architecture of Components in Root Cell Walls

**DOI:** 10.3389/fpls.2017.01882

**Published:** 2017-11-08

**Authors:** Xiuwen Wu, Muhammad Riaz, Lei Yan, Chenqing Du, Yalin Liu, Cuncang Jiang

**Affiliations:** College of Resources and Environment, Huazhong Agricultural University, Wuhan, China

**Keywords:** boron, cell wall, pectin, ultrastructure, component

## Abstract

Boron (B) is a micronutrient indispensable for citrus and B deficiency causes a considerable loss of productivity and quality in China. However, studies on pectin composition and architecture of cell wall components in trifoliate orange roots under B deficiency condition are not sufficient. In this study, we investigated the alteration in pectin characteristics and the architecture of cell wall components in trifoliate orange [*Poncirus trifoliata* (L.) Raf.] roots under B starvation. The results showed that B-deficient roots resulted in a significant enlargement of root tips and an obvious decrease in cell wall B and uronic acid content in Na_2_CO_3_-soluble pectin compared with B-adequate roots. Meanwhile, they showed a decrease of 2-keto-3-deoxyoctanoic acid in CDTA-soluble and Na_2_CO_3_-soluble pectin in cell walls, while the degree of methylation (DM) of CDTA-soluble pectin was significantly increased under B deficiency. Transmission electron microscope (TEM) micrographs of B deficient plants showed a distinct thickening of the cell walls, with the thickness 1.82 times greater than that of control plant roots. The results from Fourier-transform infrared spectroscopy (FTIR) showed that B deficiency changed the mode of hydrogen bonding between protein and carbohydrates (cellulose and hemicellulose). The FTIR spectra exhibited a destroyed protein structure and accumulation of wax and cellulose in the cell walls under B starvation. The ^13^C nuclear magnetic resonance (^13^C-NMR) spectra showed that B starvation changed the organic carbon structure of cell walls, and enhanced the contents of amino acid, cellulose, phenols, and lignin in the cell wall. The results reveal that the swelling and weakened structural integrity of cell walls, which induced by alteration on the network of pectin and cell wall components and structure in B-deficient roots, could be a major cause of occurrence of the rapid interruption of growth and significantly enlarged root tips in trifoliate orange roots under B-insufficient condition.

## Introduction

Boron (B) is an essential micronutrient for higher plants. The deficiency of boron causes various growth defects mainly in the young and growing parts of plants (Loomis and Durst, 1992). Conclusive evidence shows that B deficiency is a widespread problem than any other micronutrient (Shorrocks, 1997). Boron deprivation causes a wide range of physiological and biochemical changes in allocation of B in cell walls, pectin composition, cell wall components and structure (Hu and Brown, 1994; Pan et al., 2012; Liu et al., 2013, 2014).

Several studies have demonstrated that B deficiency results in the swelling of the cell wall and changes in the cell wall polysaccharides, and B deficiency induces increase of cell wall porosity (Fleischer et al., 1999; Ishii et al., 2001; Kaku et al., 2002). The primary function of B is involved in the formation of cell walls through diester bridges between two rhamnogalacturonan II (RG-II) molecules to cross-link cell wall pectin (Ishii and Matsunaga, 1996; Kobayashi et al., 1996; O’Neill et al., 2001). Pectic polysaccharides are known to contribute to the mechanical strength and physical properties of primary walls by binding to B (Ridley et al., 2001). Matoh et al. (1996) provided evidence that RG-II is the unique binding site of B in most plants, and B concentration in cell walls was positively correlated to the characteristic sugar residue [2-keto-3-deoxyoctanoic acid (KDO)] of RG-II. It is widely accepted that pectin is synthesized in the Golgi and then secreted into the walls as highly methyl-esterified forms. The variation in the degree of methylation (DM) of pectin after releasing carboxyl groups leads to an alteration in the binding capacity of B (OH) in the synthesis of the cell wall structure (Kobayashi et al., 1996; O’Neill et al., 1996). Thus, DM of pectin determines the integrity of cell walls.

Citrus is one of the most important economic crops in China, and B deficiency is frequently observed in citrus orchards, causing loss of productivity and quality (Han et al., 2008; Jiang et al., 2009). Recent studies showed that B deficiency resulted in the alteration of citrus root morphology, physiological characteristics and apical subcellular structure (Lu et al., 2014; Zhou et al., 2014; Liu L.C. et al., 2015). Various studies have been carried out on the changes induced by B deficiency in the cellular structures of root tips and leaves in citrange (Liu L.C. et al., 2015), the structure and ultrastructure of roots and leaves in citrus (Mesquita et al., 2016), cellular B allocation and pectin composition in leaves of two citrus rootstocks and the architecture of cell wall components in navel orange leaves (Liu et al., 2013, 2014). Furthermore, the growth characteristics generally vary greatly between roots and leaves as well as rootstocks and grafted seedlings. Trifoliate orange [*Poncirus trifoliata* (L.) Raf.] is considered the most important rootstock of citrus, and is very sensitive to B deficiency. Root morphology and growth status have an important influence on the absorption of water and nutrients, which restrict the productivity and quality of citrus. Roots are the most sensitive organ to B deficiency that results in the rapid inhibition of growth with a significantly enlarged root tip (Bohnsack and Albert, 1977). Recently, several have studied the root morphology, root vessel anatomy, metabolite profile and metabolic pathway under B deficiency condition (Wang R.D. et al., 2013; Liu G.D. et al., 2015; Dong et al., 2016).

Fourier-transform infrared spectroscopy (FTIR) and nuclear magnetic resonance (NMR) are important and appropriate techniques for analysis of the chemical composition of biological macromolecules and for identifying the structure of organic compounds dynamics (Griffiths and Haseth, 2007; Li et al., 2009; Wang Q.J. et al., 2013).

Although B deprivation has been frequently reported to induce obvious changes in the cell wall pectin composition and the architecture of cell wall components in the leaves of trifoliate orange rootstock seedlings with inadequate B. However, studies on the roots of trifoliate orange are not enough, especially in pectin composition of cell walls.

The aim of this study was to investigate (1) the changes in the pectin characteristics, exactly variations on KDO and DM in the B-deficient roots (2) the variations in the structure of organic compounds and components of root cell walls by the technique of trifoliate orange by method of transmission electron microscope (TEM), FTIR, and ^13^C-NMR, and to gain a new insight into the mechanism of B in the root cell walls of trifoliate orange.

## Materials and Methods

### Plant Material and Treatments

The experiment was carried out in a greenhouse under natural sunlight conditions at Huazhong Agricultural University, Wuhan, China. Young plants of trifoliate orange [*Poncirus trifoliate* (L.) Raf.] rootstock with uniform root length (5–6 cm) and stem height (6–7 cm) were collected and grown in the hydroponics for 9 weeks. The modified Hoagland and Arnon (1950) solution was used as a nutrient culture solution containing the following macronutrients: 2 mM KNO_3_, 1.23 mM Ca(NO_3_)_2_, 0.5 mM MgSO_4_, 0.14 mM Na_2_HPO_4_, 0.32 mM NaH_2_PO_4_, 9.15 μM MnCl_2_, 1.6 μM ZnSO_4_, 0.32 μM CuSO_4_, 0.36 μM Na_2_MoO_4_, and 37.4 μM Fe-EDTA. After being soaked in tap water for 2 days, plants were transplanted to 4-liter black plastic barrels with a nutrient solution with varying concentrations of B: 10 μM H_3_BO_3_ (control treatment) and 0 μM H_3_BO_3_ (B deficiency treatment). The experiment was designed in a completely randomized with two treatments, and each treatment was replicated six times with one replication contained one seedling.

The culture solution was aerated for 20 min at a 4-h interval and renewed once a week. Analytical-grade reagents were used to prepare nutrient solutions. The pH was maintained between 5.8 and 6.2 every day using 0.5 M H_2_SO_4_ or 1 M NaOH.

### Plant Sampling and Boron Analysis

At the end of the experiment, all plants were harvested and rinsed in deionized water, then were divided into separate parts (roots, stems, and leaves). All the roots of each seedling were further separated into two portions, with one portion used for B analysis, and the other portion used for subcellular structure observation and extraction of cell walls.

For analysis of B contents, the root samples were dried in an oven at 75°C until a constant weight. The samples were ground to a fine powder after measuring the dry weight, and washed at 500°C for 5 h, followed by dissolving the ashes in 10 mL 0.1 M HCl. The B concentration was measured spectrophotometrically at 540 nm (Hitachi UV-3100 UV/VIS; TECHCOMP, Shanghai, China) by the curcumin colorimetric method.

### Preparation of Cell Wall Materials (CWM)

The cell walls were extracted from the fresh roots of trifoliate orange by using the method described by Hu and Brown (1994). Briefly, fresh root samples were homogenized in liquid nitrogen with a mortar. After homogenization with 30 mL ice-cold ultrapure water and centrifugation at 5,000 × *g* for 10 min, the precipitate was washed with 30 mL ice-cold ultrapure water and re-centrifuged. Then the residue was washed three times with 30 mL 80% ethanol and once with 30 mL mixture of methanol/chloroform (1/1, v/v). Finally, the precipitate was washed with 30 mL acetone. The final insoluble pellet was defined as CWM. The CWM samples were dried in a freeze drier (SIMfreeze-drierFD5-3, Beckman) and weighed. The obtained CWM was divided into three portions: one portion was dried to ashes at 500°C for B determination following the procedure above, another portion was used for fractionation and determination of cell wall pectin, and the third portion was used for FTIR and ^13^C-NMR analysis.

### Preparation of Pectin

Pectin was extracted as described by Redgwell and Selvendran (1986) with slight modifications. Briefly, the crude cell wall powder was suspended in 0.05 M sodium acetate buffer (pH 6.5) containing 0.05 M CDTA. After being stirred for 12 h at 24°C in a horizontal shaker, the supernatant was defined as CDTA-soluble pectin fraction. The CDTA-insoluble pellet was then re-suspended in 0.05 M Na_2_CO_3_ and incubated for 12 h at 24°C. After centrifugation at 5,000 × *g* for 20 min at 4°C, the supernatant was designated as Na_2_CO_3_-soluble pectin fraction.

### Determination of Uronic Acid, 3-Deoxy-d-manno-2-octulosonic Acid (KDO), and the Degree of Methylation (DM) of Pectin

The contents of uronic acid (UA) in CDTA-soluble and Na_2_CO_3_-soluble pectin fractions were determined by the method of Blumenkrantz and Asboe-Hansen (1973). A standard curve was constructed with galacturonic acid and measurements were done in quadruplicate.

The level of KDO was measured by the thiobarbituric acid method (York et al., 1985) with KDO as the standard.

The DM of the two different pectin fractions was determined following the method of Anthon and Barrett (2004) with minor modifications. Briefly, the mixture of 100 μL of pectin extract and 50 μL of 1.5 M NaOH was treated for 30 min at 25°C, followed by the addition of 55 μL (0.75 M) H_2_SO_4_, 200 μL of 0.2 M Tris-HCl (pH 7.5), 80 μL of 3 mg mL^-1^ MBTH and 20 μL of alcohol oxidase (AO, 0.01 units μL^-1^) and then incubation for 20 min at 30°C. The reaction was terminated by the addition of 400 μL of solution containing 5 mg mL^-1^ of ammonium ferric sulfate and sulfaminic acid. Finally, after standing for 20 min at room temperature, the reaction mixture was supplemented with 1,095 μL of water and the absorbance was measured at 620 nm by a spectrophotometer.

### Preparation of Transmission Electron Microscope (TEM) Slices

The preparation TEM slices were carried out by the method of Kong et al. (2013) with slight modification. Briefly, the root tips were embedded in paraffin and cut into small pieces. The samples were fixed in 2.5% glutaraldehyde in phosphate buffer solution (PBS) for 12 h at 4°C. The samples were post-fixed in 1% buffered osmium tetroxide for 2–3 h and dehydrated using a mixture of 90% ethanol and 90% acetone for 15 min. Ultrathin sections were stained with 2% uranyl acetate and lead citrate, and were examined with a TEM (Hitachi 500 electron microscope) at an acceleration voltage of 60 kV. For each treatment, at least four plants were analyzed and representative plant images were chosen for each B treatment. The cell wall thickness was measured with the “Ruler” tool in Adobe Photoshop CS6 by comparing with the scale in TEM images.

### Analysis of Composition and Structure of Cell Walls by Fourier-Transform Infrared Spectroscopy (FTIR)

A small amount of cell wall powder was mixed uniformly with KBr (1/100, m/m) and pressed into tablets. IR spectra (4,000–400 cm^-1^) were recorded using a VERTEX 70 spectrometer with a resolution of 4 cm^-1^ and 32 scans per sample. The six copies of the spectra of cell walls with different B treatments were normalized and baseline-corrected with OMNIC 32 software, and graphical data were processed with Origin 8.6 software.

### Solid-State Nuclear Magnetic Resonance (^13^C-NMR) Spectroscopy Analysis

The cell walls were ground to fine powder and passed through a 0.2 mm sieve. The ^13^C-NMR spectra were obtained on a fully automatic nuclear magnetic resonance spectrometer (Bruker Avance III 400) using a 4 mm magic angle probe at 100.63 MHz. The cross–polarization/total suppression of sidebands (CP/TOSS) NMR spectrum was recorded with a 1 s recycle delay and 4,096 scans.

### Statistical Analysis

Graphs were prepared by Microsoft Excel 2010 and Origin 8.6 software. FTIR spectra were normalized and baseline-corrected with OMNIC 32 software, and then patterns were exported using Origin 8.6. The ^13^C-NMR data were analyzed with Topspin 3.2 and then plotted with Adobe Illustrator CS5. The data were statistically analyzed by SAS 9.1.3 software. Unless otherwise noted, results were presented as mean ± SD of six replicates. Significant differences (*P* < 0.05) among treatments were determined by Tukey test and significant differences (*P* < 0.05) within each group were indicated by different lower case letters (a, b).

## Results

### Plant Growth and Dry Mass Accumulation

**FIGURE 1:**
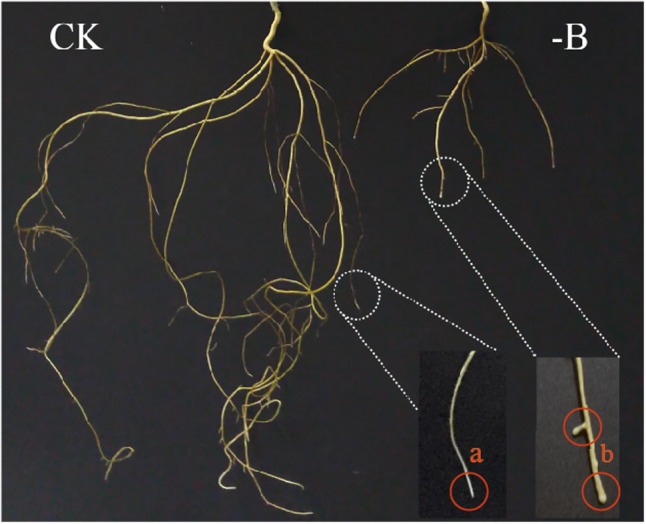
Growth of trifoliate orange roots under different boron treatments (CK: 10 μM B; -B: 0 μM B). (a) Control plant: normal healthy root growth with longer main and lateral roots; (b) boron-deficient roots: stunted root growth with thickened root tips and fewer lateral roots.

The treatment of 9 weeks without B supply showed significant inhibition on the growth of trifoliate orange roots and swollen root tips. The plants exposed to B deprivation stress exhibited shorter root length and fewer lateral roots as compared with the control treatment (adequate B, 10 μM) (**Figure 1**). The length of B-starved root and shoot decreased 64.86% and 46.35%, respectively (**Table 1**). Additionally, B deficiency remarkably reduced the dry mass accumulation of roots, stems, and leaves compared with the control treatment.

**Table 1:** Effect of boron deficiency on plant growth and dry weight of trifoliate orange seedlings.

Treatment	Root length (cm/plant)	Shoot length (cm/plant)	Dry weight (g/plant)		
			Root	Stem	Leaf
CK	40.70^a∗^ ± 3.20	20.80^a^± 0.90	0.34^a^± 0.01	0.25^a^± 0.02	0.26^a^± 0.02
-B	14.30^b^± 1.27	11.16^b^± 0.64	0.16^b^± 0.01	0.17^b^± 0.01	0.10^b^± 0.01

*^∗^Values are means of four replicates ±SD. Columns with different letters (a, b) are significantly different using the *T*-test (*n* = 6, *P* < 0.05).*

### Distribution of Boron in Roots and Cell Walls

The results presented in **Table 2** showed that B concentration in roots and cell walls were significantly decreased under B deprivation compared with the control. In B-deficient trifoliate orange seedlings, the proportion of cell wall B in root B was much greater than that of the control. These results suggested that B in roots was assigned preferentially to cell walls under B starvation.

**Table 2:** The assignment of boron in roots and cell walls of trifoliate orange roots.

Treatment	Root B content	Cell wall B content	Cell wall B/Root B	
	mg/kg dry weight	mg/kg dry weight	%	Increase (%)
CK	14.29^a∗^± 1.12	20.29^a^± 0.72	27.45^b^± 3.26	–
-B	8.05^b^± 0.06	18.68^b^± 0.48	61.47^a^± 7.58	123.93

*^∗^Values are means of four replicates ±SD. Columns with different letters (a, b) are significantly different using the *T*-test (*n* = 6, *P* < 0.05).*

### Changes in Uronic Acid, KDO, and DM of Two Different Pectins

The UA and KDO content of CDTA-soluble pectin and Na_2_CO_3_-soluble pectin in the cell wall of roots were both decreased under B deprivation treatment compared with control treatment (**Table 3**), suggesting the inhibition of the synthesis of the two different kinds of pectin and the decrease of the binding sites of B in cell walls under B deficiency. It should be noted that B deprivation had a remarkable effect on Na_2_CO_3_-soluble pectin than CDTA-soluble pectin. Additionally, B deficiency increased the DM of CDTA-soluble pectin in roots (**Table 3**). The results suggested that the reduction of pectin and B binding sites induced by B deficiency hindered the binding of B to cell wall, and the toughness of cell wall was destroyed because of the increase of DM of CDTA-soluble pectin.

**Table 3:** Effect of boron deprivation on uronic acid, KDO, and DM of CDTA-soluble pectin and Na_2_CO_3_-soluble pectin in cell wall.

	CDTA-soluble pectin		Na_2_CO_3_-soluble pectin	
	CK	-B	CK	-B
Uronic acid %	6.42^a∗^ ± 0.25	5.22^a^± 0.15	22.06^a^± 0.59	14.84^b^± 1.67
KDO%	8.79^a^± 0.79	6.92^b^± 0.54	13.66^a^± 1.60	9.30^b^± 0.25
DM%	17.30^b^± 1.18	31.88^a^± 2.18	15.40^a^± 0.50	16.35^a^± 1.34

*^∗^Values are means of four replicates ±SD. Rows with different letters (a, b) are significantly different using the *T*-test (*n* = 6, *P* < 0.05).*

### Analysis of Subcellular Structure, Thickness, and Extraction Ratio of Cell Wall of Roots

**FIGURE 2:**
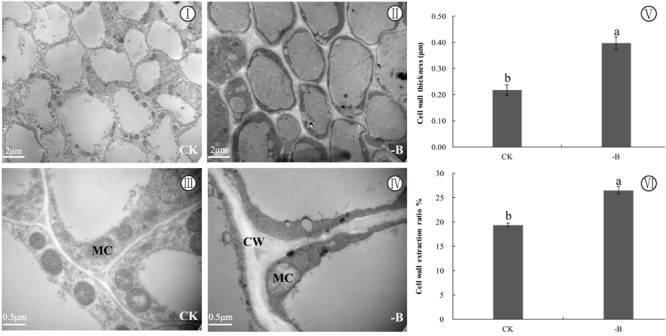
Changes in subcellular structure and cell wall under two different boron treatments (CK: 10 μM B; -B: 0 μM B). Explanation of plate (**I, II, III, IV**: TEM micrographs of root cell; **V**: cell wall thickness; **VI**: cell wall extraction ratio; CW, cell wall; MC, mitochondrion). Bars represent means of four replicates ±SD. Different letters in each group indicate significant differences at the 5% probability level. Cell wall extraction ratio % = Dry weight of cell wall materials (g)/Dry weight of roots used for cell wall extraction (g) × 100%. Different letters (a, b) indicate the significant difference using the *T*-test (*n* = 6, *P* < 0.05) between the two different boron treatments.

Transmission electron microscope micrographs of the root tips showed a thickened cell wall in cells under B-deficient treatment (**Figures 2II,IV**) while the cell wall of root tips under normal condition was regular (**Figures 2I,III**). And B deficiency significantly resulted in the increase of cell wall extraction ratio (**Figure 2VI**). Additionally, there were fewer mitochondria in cells of B-starved roots. The cell wall thickness measured by Photoshop CS6 of B-deprived root tips was remarkably greater than that of the control (**Figure 2V**).

### Changes in Composition and Structure of Cell Wall of Roots

**FIGURE 3:**
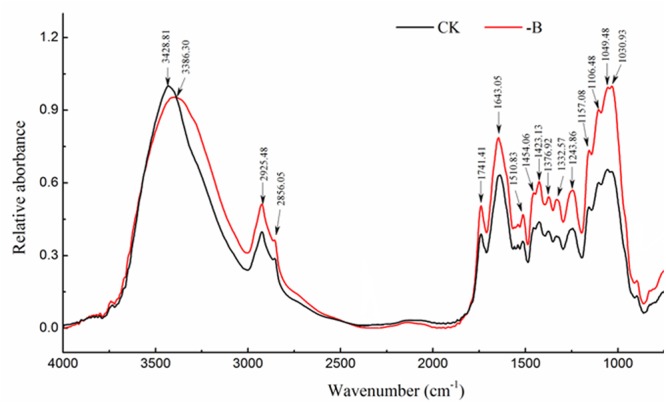
FTIR spectral analysis of changes in the composition and structure of cell walls with different boron treatments (CK: 10 μM B; -B: 0 μM B).

In the present study, the differences of characteristic peaks between CK and -B were mainly observed in the region of 4,000–800 cm^-1^. The result showed that the relative absorbance corresponding to characteristic peaks of cell wall in B-deficient roots was higher than that in control roots (**Figure 3**).

As shown in Supplementary Table 1, the peak located around 3,430 cm^-1^ corresponded to O-H and N-H stretching vibrations and mainly related to protein and carbohydrates (Yang and Yen, 2002). B starvation resulted in the obvious shift from 3,428.81 to 3,386.30 cm^-1^ was observed in cell walls, suggesting that B deficiency weakened the hydrogen bond between protein and carbohydrates (cellulose, hemicellulose). The higher intensity of 2,925 and 2,856 cm^-1^ in B-deficient spectra suggested the accumulation of wax and cellulose in cell walls. Moreover, spectra of cell walls from B-deficient roots had greater relative absorbance at ∼1,740 cm^-1^, which is characteristic of the C=O stretching vibration of alkyl-esters in pectin. The peak at 1,643 and 1,510 cm^-1^ corresponded to amide I and amide II, respectively, while 1,330 and 1,245 cm^-1^ were attributed to C-N stretching and N-H deformation from amide III, respectively. Changes in those related peaks indicated that B starvation destroyed the protein structure of root cell walls. The enhanced intensity of 1,420 cm^-1^ was attributed to –COO^-^ stretching in B-deficient cell walls, implying the increase of some amino acids. In addition, B deficiency significantly increased the relative concentration of cellulose as indicated by the enhanced characteristic peaks of cellulose (1,454, 1,376, 1,050, and 1,030 cm^-1^) (**Figure 3**).

### Changes in Organic Carbon of Cell Walls of Roots

**FIGURE 4:**
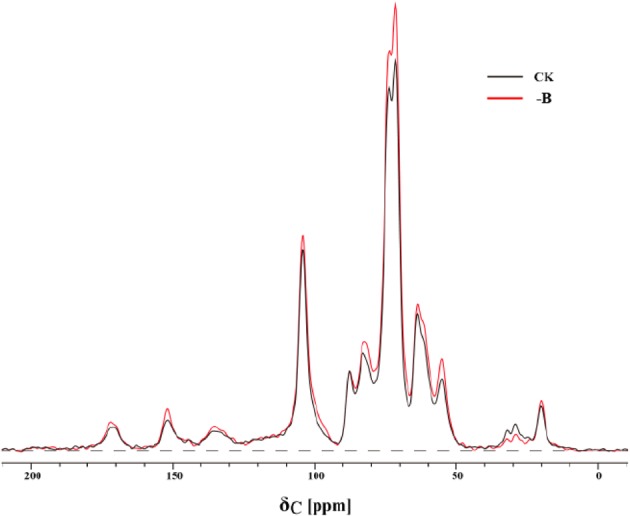
^13^C-NMR spectra in cell walls of roots under normal boron supply (CK: 10 μM B) and boron deficiency (-B: 0 μM B).

The ^13^C-NMR CP/TOSS spectra of cell walls could be divided into 8 resonance regions as shown in Supplementary Table 2 and **Figure 4**. The aliphatics (0–112 ppm) consisted of the alkyls C, the methoxyl C, the carbohydrates C and the di-o-alkyl C, while the aromatics (112–160 ppm) was associated with the aryl C and the phenolic C.

The variations in the organic carbon of cell walls under B-deficient and B-adequate conditions were observed (**Table 4**). Specifically, the organic carbon of cell walls of roots was dominated by aliphatics in the two groups. B deprivation increased the signals of OCH_3_ groups and OCHO groups, but decreased the signals of C_αα_-OR groups. Additionally, B deficiency induced obvious changes in aromatics C, which was mainly reflected in C-O. The variation in those integrations suggested that B starvation changed the organic carbon structure of cell walls of roots, increased the contents of amino acids, cellulose, phenols and lignin in cell walls, and reduced the content of carbohydrate, mainly the soluble sugar.

**Table 4:** The relative content (%) of different types of organic carbon in different resonance regions (ppm) of cell walls of roots under two different boron treatments (CK: 10 μM B; -B: 0 μM B).

Treatment	0–45	45–62	62–92	92–112	112–121	121–141	141–160	160–190	0–112	112–160
	CH_3_	O-CH_3_	C_α_-OR	OCHO	C-H	C-C	C-O	COO	Aliphatics	Aromatics
CK	4.47^a∗^± 0.23	10.27^b^± 0.14	58.22^a^± 2.12	16.07^b^± 1.02	1.55^a^± 0.05	3.29^a^± 0.12	3.17^b^± 0.27	2.56^a^± 0.19	89.23^a^± 4.58	8.00^b^± 0.29
-B	4.56^a^± 0.21	11.18^a^± 0.12	57.32^b^± 2.56	16.68^a^± 0.96	1.60^a^± 0.11	3.43^a^± 0.16	3.74^a^± 0.35	2.61^a^± 0.21	88.74^a^± 7.25	8.76^a^± 0.51

*^∗^Values are means of four replicates ±SD. Columns with different letters (a, b) are significantly different using the *T*-test (*n* = 6, *P* < 0.05).*

## Discussion

It has been reported that B is required for the structural integrity of cell walls (O’Neill et al., 2001). The present study on trifoliate showed that B-deficient plants resulted in significant inhibition of root elongation and thickness root tips. Moreover, B-starved roots exhibited remarkably fewer mitochondria in the cells and dramatically increased of cell wall thickness and cell wall materials. Boron may affect the extensibility of cell walls not only via cross-linking of B-RG-II (O’Neill et al., 2001) but also by affecting the distribution of pectins cross-linked with calcium (Ca) (Matoh and Kobayashi, 1998). Previous study have indicated that B and Ca cross-linked pectins can be internalized via endocytosis in root apex cells, and their internalization might prove to be of critical importance for growth, polarity, and morphogenesis of roots (Baluska et al., 2002, 2005; Dhonukshe et al., 2006). Under B deprivation, abundance of pectins rapidly increased in cell walls of maize and wheat (insensitive to B deficiency) root apices because of the inhibition on their internalization, whereas root cell walls of species sensitive to B deprivation, like zucchini and alfalfa, did not show accumulation of pectins and internalization of cell wall pectins under B deficiency (Yu et al., 2002). Besides, aluminum (Al) toxicity in several aspects also thickened cell walls and inhibited endocytosis of cell wall pectins (Shen et al., 2008; Wu et al., 2015), and supply of B has been proved to alleviate Al toxicity in higher plants, possibly via cell wall pectins (Stass et al., 2007; Zhou et al., 2015). In the present study, B deficiency decreased the UA contents and KDO (the characteristic sugar residue of RG-II) of CDTA-soluble pectin and Na_2_CO_3_-soluble pectin of trifoliate orange roots, suggesting that B binding sites in cell walls were reduced and the network of pectin was modified by B starvation. Additionally, B deprivation significantly increased the DM of CDTA-soluble pectin, which may weaken the structure of cell walls in trifoliate orange roots. Consistent with this, the intensity of absorbance at 1,736 cm^-1^ was higher in B-deficient root cell wall, indicating the strengthening of the C=O stretching vibration of alkyl-esters in pectin. It is worth mentioning that pectic polysaccharides influence the expansion, thickness and porosity of the cell walls and B-RG-II compounds in cell walls provide mechanical strength to the cell wall and structure (O’Neill et al., 2001). Therefore, alteration on distribution patterns and properties of pectin in cell walls may destroy the integrity of cell walls in trifoliate orange roots.

Hydrogen bonding is the most prevalent bonding type between the macromolecules in the cell walls. A large number of hydrogen bonds contribute to establishing a tight but flexible connection between macromolecules as the bonds can be easily opened and reformed due to their low bonding strength (Burgert and Dunlop, 2011). FTIR spectra from cell walls of B-starved roots exhibited a remarkable shift to the lower frequency corresponded to O-H and N-H stretching vibrations and are associated with mainly protein and carbohydrates, indicating that B deficiency destroyed the linkage pattern between protein and carbohydrates. In addition, the decrease of some corresponding peaks in cell walls indicates the inhibition of the protein synthesis and showed destroyed cell walls structure due to B starvation. The ^13^C-NMR spectra further revealed the increased relative content of methoxyl C associated with amino acids. The cell walls contained a variety of wall-associated proteins, and the structural proteins supports in the mechanical strength of the wall and facilitate the proper assembly of other wall components (Jamet et al., 2006).

Boron deficiency not only increased the amount of cellulose but also changed its architecture in cell walls. Similar results were also obtained in leaf cell walls of a navel orange (Liu et al., 2014) and roots of rape (Yang and Yen, 2002). It has been reported that the *myo*-inositol oxidation pathway plays a very important role in cell wall polysaccharide (cellulose) biosynthesis (Loewus and Murthy, 2010), and a significant decrease was observed in the *myo*-inositol concentration of B-starved leaves of navel orange (Liu G.D. et al., 2015; Dong et al., 2016). Thus the increase of cellulose may be related to changes induced by B deprivation in the *myo*-inositol oxidation pathway. As a complex macromolecule covalently, lignin plays an essential role in linking polysaccharides in plant cell walls and performing important biological functions (Tzin and Galili, 2010). It has been suggested that B could participate in lignin metabolism (Bellaloui, 2012) and lignin biosynthesis was obviously increased in trifoliate orange roots under B-deprived conditions (Dong et al., 2016). Our ^13^C-NMR spectral results showed higher relative contents of aryl C and phenolic C from lignin under B deprivation, suggesting a prominent promoting effect of B deficiency on lignin biosynthesis in cell walls of roots.

Phenolic compounds, generated from pentose phosphate pathway (PPP), are important secondary metabolites in plants. B starvation obviously promoted accumulation of phenols in cell walls of roots due to the entry of substantial amounts of respiratory substrates into PPP. Furthermore, accumulation of phenols caused a rapid rise of polyphenol oxidase activity, thereby resulting in the production of a large amount of active quinone and oxyradical (Shkol’Nik et al., 1981). Dordas and Brown (2005) suggested that the increase of phenols due to B deficiency be a secondary effect on the death of cells.

**FIGURE 5:**
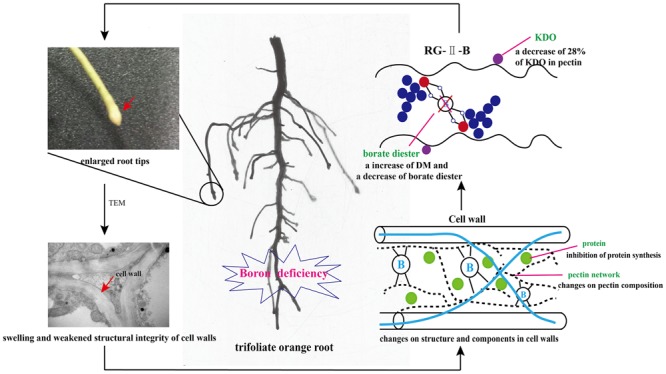
Schematic diagram in the changes of cell walls in trifoliate orange roots due to boron (B) deficiency stress. Explanation of plate (KDO, 2-keto-3-deoxyoctanoic acid; DM, the degree of methylation of pectin).

Changes in the structure of cell walls were correlated with the destruction of hydrogen bonding between carbohydrates/protein and network structure of pectin, and higher lignin in cell walls aggravated suberification in roots without adequate B. These results suggest that the changes induced by B starvation in the composition and structure of cell walls determine, to some extent, B deficiency symptoms of roots. A schematic summary, describing the key responses on pectin characters, cell wall structure and components in B-deficient trifoliate orange roots, and their relationships between the occurrence of B starvation symptoms of roots and changes on cell walls, is proposed in **Figure 5**.

## Conclusion

B deficiency induced a decrease in the Na_2_CO_3_-soluble pectin content in root cell walls and inhibited the synthesis or secretion of RG-II in pectin. Meanwhile, the DM of CDTA-soluble pectin was increased in the cell walls of B-deprived roots, and weakened the cell wall structural integrity. Additionally, B deficiency influenced the hydrogen bond between protein and carbohydrates (cellulose, hemicellulose) and promoted the accumulation of cellulose and phenolic compounds in cell walls. Therefore, the alteration in the architecture of cell wall components such as pectin, protein and cellulose, as well as the linkage pattern among them is closely related to the reduced capabilities of B binding. The results contribute to a better understanding of the mechanism of B in root cell walls and the root B deprivation symptoms, especially in the fields of rootstocks.

## Author Contributions

XW and CJ designed and supervised this study; XW conducted the experiments, performed data interpretation, and drafted the manuscript; LY and CD helped replace nutrition solution in the experiment and determine B concentration; YL helped revise the manuscript in grammar. All authors read and approved the final manuscript.

## Conflict of Interest Statement

The authors declare that the research was conducted in the absence of any commercial or financial relationships that could be construed as a potential conflict of interest.
