# Supplementary material for: Boron Deficiency in Trifoliate Orange Induces Changes in Pectin Composition and Architecture of Components in Root Cell Walls
Source: Front Plant Sci. 2017 Nov 8;8:1882. doi: 10.3389/fpls.2017.01882 (PMC5682329; doi:10.3389/fpls.2017.01882)
Supplement: Supplementary file 1 [file Table_1.doc]

**Supplementary Table 1** The assignment of absorption bands to their major chemical components in cell walls of roots under normal boron supply (CK: 10 μM B) or boron deficiency (-B: 0 μM B)

| Number | Wavenumber (cm^-1^) | Functional group | Major component |
| --- | --- | --- | --- |
| 1 | 3430 | -OH, N-H stretching | protein, carbohydrate (cellulose, hemicellulose) |
| 2 | 2925 | C-H stretching | wax, cellulose |
|  | 2856 |  |  |
| 3 | 1740 | C=O stretching from -COOR | pectin |
| 4 | 1643 | C=O stretching from -CO-NH- | amideⅠ |
| 5 | 1510 | C-N stretching and N-H deformation | amideⅡ |
| 6 | 1454 | C-H deformation | cellulose |
| 7 | 1420 | -COO**^-^** stretching | amino acid, cellulose |
| 8 | 1376 | C-H stretching | cellulose |
| 9 | 1330 | C-N stretching and N-H deformation | amide Ⅲ |
|  | 1245 |  |  |
| 10 | 1155 | C-O stretching | amino acid residues, cellulose |
| 11 | 1105 | C-C, C-O stretching | carbohydrate chain from cellulose |
|  | 1050 |  |  |
|  | 1030 |  |  |

**Supplementary Table 2** The assignment of major chemical shift of organic carbon in cell walls of roots under normal boron supply (CK: 10 μM B) or boron deficiency (-B: 0 μM B)

| Chemical shift (ppm) | Assignment | Major component |
| --- | --- | --- |
| 0-45 | Alkyl C | wax, cuticle |
| 45-62 | Methoxyl C | amino acid |
| 62-92 | Carbohydrate C | carbohydrate |
| 92-112 | Di-O-alkyl C | cellulose |
| 112-121 | Aryl C | polyphenol, lignin |
| 121-141 |  |  |
| 141-160 | Phenolic C | phenols, lignin |
| 160-190 | Carboxyl C | carboxyl |
